# Targeted Expression of Myelin Autoantigen in the Periphery Induces Antigen-Specific T and B Cell Tolerance and Ameliorates Autoimmune Disease

**DOI:** 10.3389/fimmu.2021.668487

**Published:** 2021-06-02

**Authors:** Shin-Young Na, Gurumoorthy Krishnamoorthy

**Affiliations:** Research Group Neuroinflammation and Mucosal Immunology, Max Planck Institute of Biochemistry, Martinsried, Germany

**Keywords:** experimental autoimmue encephalomyelitis, tolerance, autoimmunity, multiple sclerosis (MS), MOG (myelin oligodendrocyte glycoprotein)

## Abstract

There is a great interest in developing antigen-specific therapeutic approaches for the treatment of autoimmune diseases without compromising normal immune function. The key challenges are to control all antigen-specific lymphocyte populations that contribute to pathogenic inflammatory processes and to provide long-term protection from disease relapses. Here, we show that myelin oligodendrocyte glycoprotein (MOG)-specific tolerance can be established by ectopic expression of MOG in the immune organs. Using transgenic mice expressing MOG-specific CD4, CD8, and B cell receptors, we show that MOG expression in the bone marrow cells results in impaired development of MOG-specific lymphocytes. Ectopic MOG expression has also resulted in long-lasting protection from MOG-induced autoimmunity. This finding raises hope that transplantation of autoantigen-expressing bone marrow cells as a therapeutic strategy for specific autoantigen-driven autoimmune diseases.

## Introduction

Autoimmunity results from a failure to induce tolerance of autoreactive T and B lymphocytes, and subsequent activation of these cells by a variety of mechanisms (1). The autoimmune regulator (AIRE) gene controls the expression of a subset of tissue-specific antigens within the thymus, thereby preventing the maturation of several autoreactive T cells (2). Nevertheless, autoreactive lymphocytes do escape thymic selection and form a natural component of the immune repertoire. The ability to control these autoreactive lymphocytes by re-establishing antigen-specific tolerance is the major objective for the treatment of autoimmune diseases. Over the past few decades, various antigen-specific therapeutic approaches that include delivery of self-antigens using DNA, mRNA, synthetic peptides, recombinant proteins, and antigen-coated nanoparticles have been shown to provide short-term therapeutic benefits in experimental models (3). However, induction of permanent tolerance is an ideal goal for the long-term management of autoimmune diseases.

One of the main difficulties in applying antigen-specific approaches to autoimmune diseases is the complexity of the autoreactive immune cell repertoire. Despite several decades of research, the definitive target autoantigen in the common neurological disease Multiple Sclerosis (MS) is yet to be identified. The myelin oligodendrocyte glycoprotein (MOG), a minor component of the compact myelin sheath, is now emerging as a target autoantigen in patients with specific clinical neurological dysfunction collectively termed as MOG-antibody disease, which does not meet the typical criteria for MS or other known neuroinflammatory conditions (4). MOG-antibody disease patients resemble clinically the neuromyelitis optica spectrum disorders in the predilection for relapses of optic neuritis and transverse myelitis and do not have aquaporin 4 (AQP4) autoantibodies. Serum autoantibodies against MOG are commonly found in these patients (5). Thus, MOG-specific tolerance approaches are attractive for the treatment of MOG-antibody disease patients.

The expression of tissue-specific antigens in the thymus and bone marrow plays a critical role in the induction of physiological tolerance through a negative selection process whereby high-avidity self-reactive T cells and B cells, are eliminated (6). For instance, neuronal antigen NF-M and the DM20 isoform of the myelin antigen, proteolipid protein (PLP), is known to be expressed in the thymus (7, 8). However, several tissue-specific autoantigens are not expressed in the thymus and bone marrow which leads to the escape of those self-antigen-specific T and B cells. MOG is exclusively expressed in the central nervous system (CNS) oligodendrocytes (9, 10). Although a report suggests that MOG is expressed in the thymus at the transcript level (11), incomplete tolerance to MOG was observed in experimental animals. Immunization of mice with MOG readily induces severe encephalomyelitis, suggesting that MOG-specific T cells are present in healthy mice.

Induction of immune tolerance could be achieved by resetting the immune system through autologous hematopoietic stem cell transplantation (AHSCT) (12). However, the re-emergence of autoreactive lymphocytes cannot be completely ruled after AHSCT. A solution to this problem is to introduce self-antigen into the bone marrow cells before transplantation to permanently dampen autoreactivity against a specific antigen without impairing general immunity. In this study, we have explored the hypothesis that long-term MOG-specific tolerance can be established by expressing full-length MOG in the peripheral immune system. We show that MOG expression in the peripheral immune organs results in life-long tolerance to MOG-specific CD4, CD8, and B cells. Functional MOG-specific T and B cells fail to develop in MOG-expressing transgenic mice and these mice were protected from autoimmune encephalomyelitis upon immunization. Finally, we show that transplantation of MOG-expressing bone marrow cells is sufficient to induce tolerance. Our results suggest that transplantation of bone marrow cells expressing MOG is an attractive strategy to re-establish MOG-specific immune tolerance in patients with MOG-antibody disease.

## Material and Methods

### Mice

For the generation of transgenic MOG overexpressing mice (Tg^MOG^), the entire coding region of murine MOG from mouse brain cDNA was amplified and inserted into the sites SalI and BamHI of the vector pHSE3´ containing H2-K^b^ promoter and immunoglobulin heavy chain enhancer (13). For the generation of TCR^25^ mice, we isolated a CD8^+^ T cell clone from C57BL/6 mice immunized with MOG_206-214._ TCR Vα5 (TRAV3-3*01) and Vβ9 (TRBV17*01) chains were amplified with specific primers and cloned into pHSE3´ vector. Prokaryotic DNA elements from the transgenic expression constructs were removed by XhoI digestion and the transgenic constructs were microinjected into C57BL/6 oocytes. The founder mouse that expressed the transgene was bred with C57BL/6 mice. 2D2 (14), RAG2^–/–^, IgH^MOG^ (15), and TCR^ova^ (OT-II) mice were maintained in the animal facilities of the Max Planck Institute of Biochemistry and Neurobiology, Martinsried, Germany.

### Proteins and Peptides

MOG_35-55_ (MEVGWYRSPFSRVVHLYRNGK), MOG_206-214_ (LAGQFLEEL), and Ova_323-339_ (ISQAVHAAHAEINEAGR) peptides were synthesized by BioTrend or core facility of Max Planck Institute of Biochemistry. Recombinant rat MOG was produced from E. coli as described earlier (16) and biotinylated using Biotin-X-NHS (Thermo Scientific) according to the manufacturer’s instructions.

### EAE Induction

Mice were injected s.c. at the tail base with an emulsion containing equal amounts of complete Freund’s adjuvant (CFA) (Difco) containing 5 mg/ml *Mycobacterium tuberculosis* (strain H37Ra) (Difco) and 200 µg MOG_35-55_ in PBS. 400 ng of pertussis toxin (List Biological Labs) was injected i.p. on days 0 and 2 following immunization. Clinical scores were assigned on a scale of 1 - 5 as follows: 0: healthy; 1: flaccid tail; 2: impaired righting reflex and/or gait; 3: one paralyzed hind leg; 4: both hind legs paralyzed; 5: moribund animal or death of the animal after preceding clinical disease.

### Antibodies and Flow Cytometric Analysis

The following anti-mouse antibodies were purchased from BD Biosciences, Biolegend, or eBioscience. CD4-PerCP-Cy5.5, CD8-APC, CD3-FITC, CD19-PE, B220-PerCP-Cy5.5, Vα3.2-FITC, Vβ11-PE, NK1.1-biotin, CD11c-FITC, CD11b-PE, Gr-1-biotin, CD21-FITC, CD23-biotin, CD25-PE, CD44-FITC, CD62L-APC, CD69-FITC, Vb9-PE, CD86-PerCP-Cy5.5, PD-1-PE, IgM^b^-FITC, IgM^a^-PE, B220-BV605, Foxp3-FITC, TCR Vβ screening panel labeled with FITC. Erythrocyte depleted single-cell suspensions were prepared from the spleen, thymus, bone marrow, and pooled lymph nodes. For the detection of cell surface markers by flow cytometric analysis, cells were stained with fluorochrome-labeled antibodies. Streptavidin-APC (BD Biosciences) was used to detect biotin-labeled antibodies. MOG expression on the cell membrane was detected with FITC labeled MOG-specific 8.18-C5 antibody. MOG-binding B cells were detected using biotinylated recombinant MOG monomer and subsequent staining with streptavidin-APC. Intracellular staining was performed according to the manufacturer’s instructions (eBioscience). Data acquisition was done with a FACSCalibur or FACSCanto system (BD Biosciences). FlowJo software (TreeStar) was used for further analysis. The general gating strategy to determine the frequencies of immune cells is shown in **Supplementary Figure 5**.

### *In Vitro* Proliferation Assay

Single-cell suspensions from spleens were prepared and 2 x 10^5^ cells/well were seeded in 96-well, round-bottom plates in a total volume of 200 µl complete RPMI medium containing 10% fetal bovine serum (Gibco). Cell were stimulated with 1 µg/ml anti-CD3 (145-2C11, BioXCell), 1 µg/ml LPS (Sigma), 50 ng/ml PMA plus 500 ng/ml ionomycin (Sigma), 2.5 µg/ml ConA (Sigma) or 20 µg/ml peptides. After a culture period of 48 hours, 1 µCi ^3^H labeled thymidine was added per well. Samples were harvested 16 hours later and thymidine incorporation was measured and represented as counts per minute (cpm). Each sample was run in triplicates. For flow cytometry-based proliferation assay, 2 x 10^7^ splenocytes were washed with PBS and incubated at 37°C for 10 min with 5 µM eFluor 450 (Thermo Fischer Scientific). Cells were cultured as above with peptides at indicated concentrations for 3 days before measurement using FACS Canto.

### Bone Marrow Transfer

Bone marrow cells were flushed from femurs of Tg^MOG^ and WT mice, and erythrocytes were removed. 5 - 10 X 10^6^ cells were injected into lethally irradiated or non-irradiated recipient mice *via* the tail vein. Immune organs were isolated and analyzed after 4 weeks.

### Adoptive Cell Transfer

Splenocytes from IgH^MOG^ mice were labeled with 5 µM eFluor 450 and 20 X 10^6^ cells were injected into Tg^MOG^ or WT littermates *via* the tail vein. The spleen cells were analyzed by flow cytometry after 5 days.

### Cytokine ELISA

Cells were plated and stimulated as described for proliferation assays. After 72 hours cell supernatants were collected. Cytokine concentrations were determined using matching antibody pairs for mouse IL-6, IL-10, IL-17, IFNγ (Pharmingen). ABTS (Sigma) was used as a color substrate and the optical density (OD) was measured at 405 nm.

### Determination of Serum Immunoglobulins

Serum collected from mice were transferred to 96-well ELISA plates (Nunc) pre-coated with respective isotype-specific capture antibodies (BD Biosciences) or recombinant MOG. After extensive washes, bound immunoglobulins were detected by a sandwich consisting of a biotinylated allotype and isotype-specific anti-mouse Ig (BD Biosciences) and a streptavidin-horseradish peroxidase (HRP) complex (BD Biosciences). ABTS (Sigma) was used as a color substrate and the OD was measured at 405 nm.

### Statistical Analysis

GraphPad Prism 9 (GraphPad Software, Inc.) was used for statistical analyses. P values below 0.05 were considered statistically significant. Bars and line graphs depict the mean ± standard error of mean.

## Results

### Expression of MOG in the Periphery Dampens MOG-Specific Immune Responses

**Figure 1:**
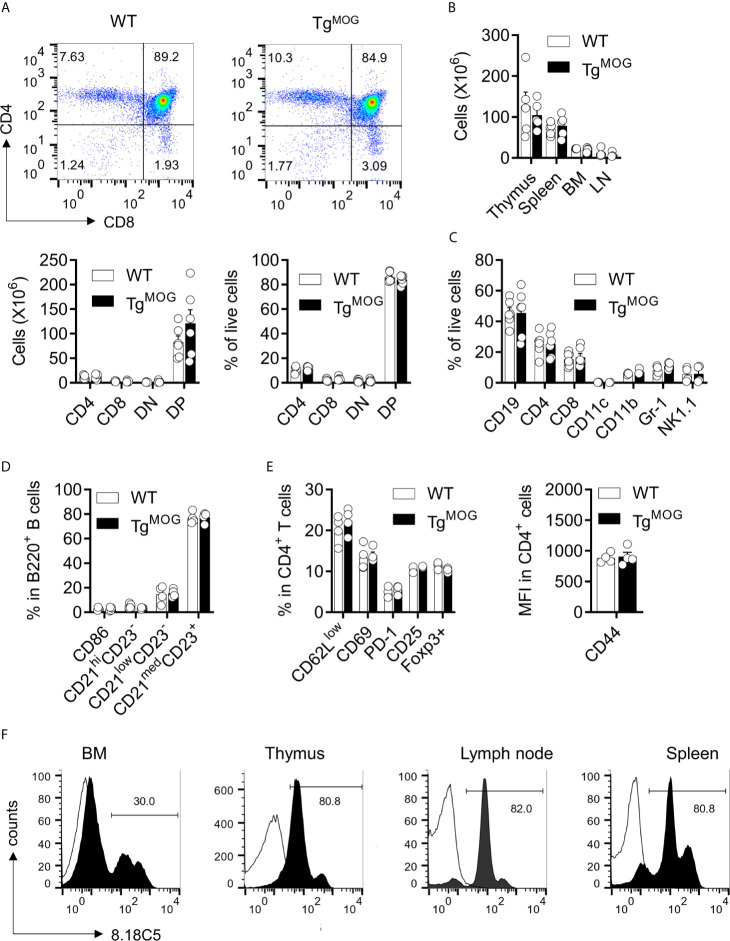
Lymphoid development in Tg^MOG^ mice. **(A)** Flow cytometry analysis of thymus from Tg^MOG^ and WT non-transgenic littermates. Representative plots are shown (top). Frequencies and total numbers of CD4^+^, CD8^+^, DN (CD4^–^CD8^–^), and DP (CD4^+^CD8^+^) T cells are depicted (bottom). **(B)** Cellularity of thymus, spleen, lymph nodes (LN), and bone marrow (BM). **(C)** Immune cell composition of the spleen from Tg^MOG^ and WT mice. Frequencies of CD19^+^, CD4^+^, CD8^+^, CD11c^+^, CD11b^+^, Gr-1^+^ and NK1.1^+^ cells in the live gate are shown. **(D)** B cell subsets in the spleen of Tg^MOG^ and WT mice. Frequencies of CD86^+,^CD21^hi^CD23^–^, CD21^low^CD23^–^, CD21^med^CD23^+^ cells in gated B220^+^ cells are shown. **(E)** Activation marker expression in splenic CD4^+^ T cells. Frequencies of CD62L^low^, CD69^+^, PD-1^+^, CD25^+^, Foxp3^+^ cells (left) or mean fluorescence intensity (MFI) of CD44 expression (right) in gated CD4^+^ cells are shown. **(F)** Representative flow cytometry histograms showing membrane MOG expression in lymphoid organs. **(A–E)** The combined data from two independent experiments is shown. n = 4 - 6 mice each group. Each circle represents an individual mouse.

We inserted a complementary DNA (cDNA) encoding MOG, a minor component of the central nervous system myelin sheath and exclusively expressed by oligodendrocytes, downstream of the H-2K^b^ promoter to allow the broader peripheral expression outside the CNS. A transgenic mouse line (hereafter referred to as Tg^MOG^) was obtained that passed the transgene stably to its offspring. The Tg^MOG^ mice had a normal life span without any abnormalities including any signs of overt autoimmunity for more than 1 year of observation. There were no differences in the thymic development of T cell subsets **(** **Figure 1A** **)**. The cellularity and the composition of immune cells in the primary and secondary immune organs, thymus, spleen, bone marrow, and lymph node were similar to non-transgenic wild type (WT) littermates **(** **Figures 1B–D**, and **Supplementary Figures 1A, C** **)**. Further, there were no differences in the activation markers CD62L, CD69, PD-1, CD25, and CD44 in CD4^+^ and CD8^+^ T cells and CD86 expression in B cells **(** **Figures 1D, E** and **Supplementary Figure 1B** **)**. Flow cytometry analysis showed the expression of MOG in the immunological organs, bone marrow, spleen, lymph node, and thymus. We particularly observed a higher MOG expression in cells of hematopoietic lineages including T and B cells in the spleen and lymph nodes **(** **Figure 1F** **)**.

**Figure 2:**
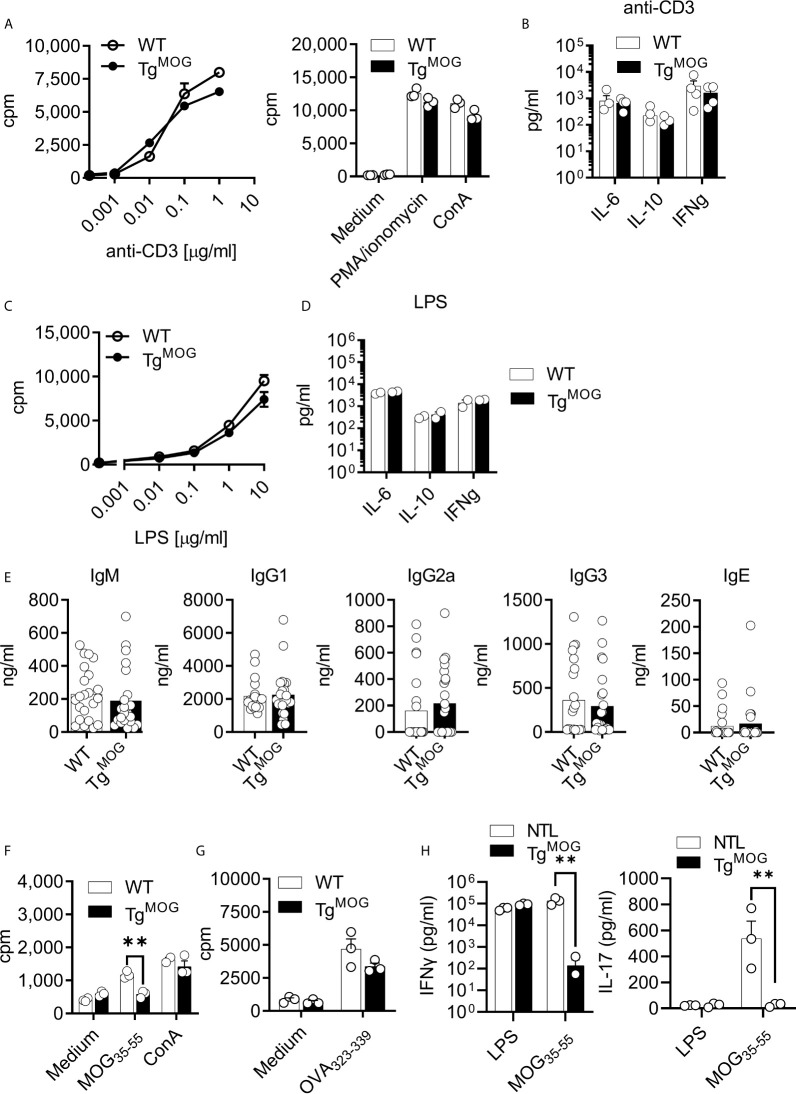
Immune responses in Tg^MOG^ mice. **(A)**, Proliferation of splenocytes from WT or Tg^MOG^ mice in response to polyclonal stimulants anti-CD3, PMA/ionomycin, and ConA. Each circle represents an individual replicate. **(B)**, Cytokine (IL-6, IL-10, and IFNγ) secretion by splenocytes in response to anti-CD3 stimulation. Each circle represents an individual mouse. n = 4 per group. **(C)**, Proliferation of splenocytes in response to LPS stimulation. **(D)**, Cytokine (IL-6, IL-10, and IFNγ) secretion by splenocytes in response to LPS stimulation. Each circle represents an individual mouse. n = 2 per group. **(E)**, Total IgM, IgG1, IgG2a, IgG3 and IgE immunoglobulin titers in the sera of Tg^MOG^ (n = 23) or WT (n = 22) mice were measured by ELISA. **(F, G)** Proliferation of splenocytes from MOG_35-55_ **(F)** or OVA_323-339_ **(G)** immunized WT or Tg^MOG^ mice. Each circle represents an individual replicate. **P = 0.0016 (Two-way ANOVA). **(H)**, Cytokine (IFNγ and IL-17) secretion of splenocytes in response to LPS or MOG_35-55_ stimulation. **P = 0.0043, **P = 0.0094 (Two-way ANOVA). n = 3 per group. **(A–D, F, G)**, Representative data from two independent experiments is shown.

We next determined the T and B cell responsiveness to polyclonal stimulants. Stimulation of splenocytes from Tg^MOG^ mice and WT littermates with anti-CD3, lipopolysaccharide (LPS), Phorbol 12-myristate 13-acetate (PMA)/ionomycin, and concanavalin A (ConA) did not show differences in proliferative response **(** **Figures 2A, C** **)**. Cytokine responses after anti-CD3 and LPS stimulation were also similar **(** **Figures 2B, D** **)**. The total serum antibody levels remained comparable between Tg^MOG^ mice and WT non-transgenic littermates **(** **Figure 2E** **)**. To study whether the transgenic expression of MOG dampens antigen-specific immune responses, we immunized Tg^MOG^ mice with either MOG_35-55_ or OVA_323-339_ and measured recall immune responses. When splenocytes from MOG-immunized Tg^MOG^ and WT non-transgenic littermates were challenged with MOG_35-55_ peptide, T cell proliferation was not detectable in Tg^MOG^ lymphocytes while remained responsive to mitogenic stimulation by ConA **(** **Figure 2F** **)**. However, immunization of Tg^MOG^ mice with ovalbumin resulted in comparable anti-ovalbumin proliferative responses similar to non-transgenic littermate **(** **Figure 2G** **)**. The recall stimulation assays also showed a reduced IFNγ and IL-17 production after MOG_35-55_ restimulation in Tg^MOG^ mice compared to WT littermates whereas LPS stimulation resulted in similar levels of IFNγ production (**Figure 2H**). Together, these data indicate that Tg^MOG^ transgenic mice have a competent immune system but remain specifically tolerant to MOG-induced immune responses.

### MOG-Expression Induces Deletion of MOG-Specific B Cells in MOG-Reactive B Cell Receptor (BCR) Transgenic Mice

**Figure 3:**
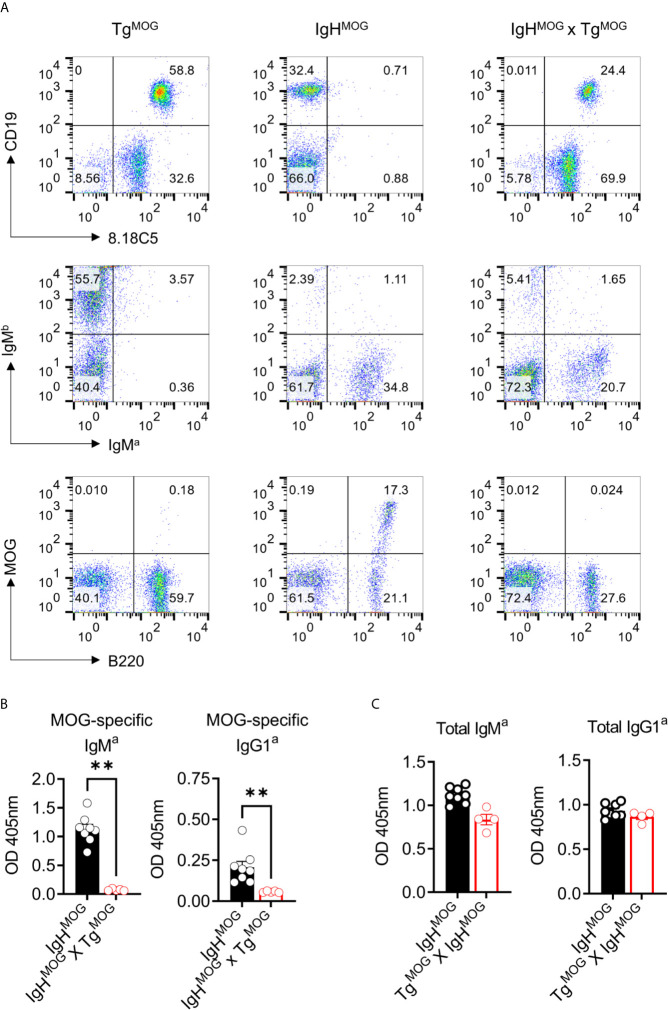
MOG-specific B cells are deleted in IgH^MOG^ X Tg^MOG^ mice. **(A)**, Representative flow cytometry data from Tg^MOG^, IgH^MOG^, IgH^MOG^ X Tg^MOG^ mice. Cells were stained with antibodies against transgenic anti-IgM^a^, WT anti-IgM^b,^ anti-MOG 8.18C5 antibodies or recombinant MOG protein along with B cell lineage markers B220 and CD19. Representative data from two independent experiments is shown. **(B, C)**, MOG-specific **(B)**, and total **(C)** IgM^a^ and IgG1^a^ antibodies in the sera were quantified by ELISA. Each circle represents an individual mouse. n = 5 – 8 mice per group. **P = 0.0016 (Mann-Whitney’s U-test, two-tailed).

MOG-specific autoimmunity is characterized by the generation of MOG-reactive autoantibodies that can induce demyelination. Therefore, we evaluated the effects of MOG expression on MOG-specific B cell response by crossing Tg^MOG^ mice with MOG-specific immunoglobulin heavy chain expressing transgenic mouse (IgH^MOG^) (15). While more than 95% of the IgH^MOG^ mice B cells express a heavy chain of a demyelinating antibody 8.18-C5, approximately about 30 – 50% of those B cells bind MOG due to random pairing of light chains from endogenous repertoire (15, 17). The expression of MOG on the cell membrane in Tg^MOG^ and IgH^MOG^ x Tg^MOG^ mice was confirmed by staining with the 8.18C5 antibody **(** **Figure 3A**; top panel). The staining of transgenic B cells with anti-IgM^a^ antibody showed a reduction in the frequencies of IgM^a^ expressing B cells and a slight elevation of IgM^b^ expressing non-trangenic B cells in IgH^MOG^ x Tg^MOG^ mice when compared to single transgenic IgH^MOG^ mice **(** **Figure 3A**; middle panel). However, few IgM^a^ expressing B cells remained in IgH^MOG^ x Tg^MOG^ mice failed to bind recombinant MOG protein **(** **Figure 3A**; bottom panel). This is in line with the fact that approximately 30 – 50% of IgH^MOG^ B cells bind MOG. Consistent with this, we observed that MOG-specific autoantibodies were also completely absent in the serum of IgH^MOG^ x Tg^MOG^ mice **(** **Figure 3B** **)**. However, we found similar levels of total IgM and IgG antibodies in the serum **(** **Figure 3C** **)**. In addition, the transfer of IgH^MOG^ mice splenocytes into Tg^MOG^ or WT littermates resulted in the deletion of MOG-specific B cells in the Tg^MOG^ recipients (**Supplementary Figure 2** **)**. Taken together, we conclude that MOG expression outside the CNS induces MOG-specific B cell tolerance by selectively deleting MOG-binding B cells.

### MOG-Expression Induces Deletion of MOG-Specific CD4^+^ and CD8^+^ T Cells in T Cell Receptor (TCR) Transgenic Mice

**Figure 4:**
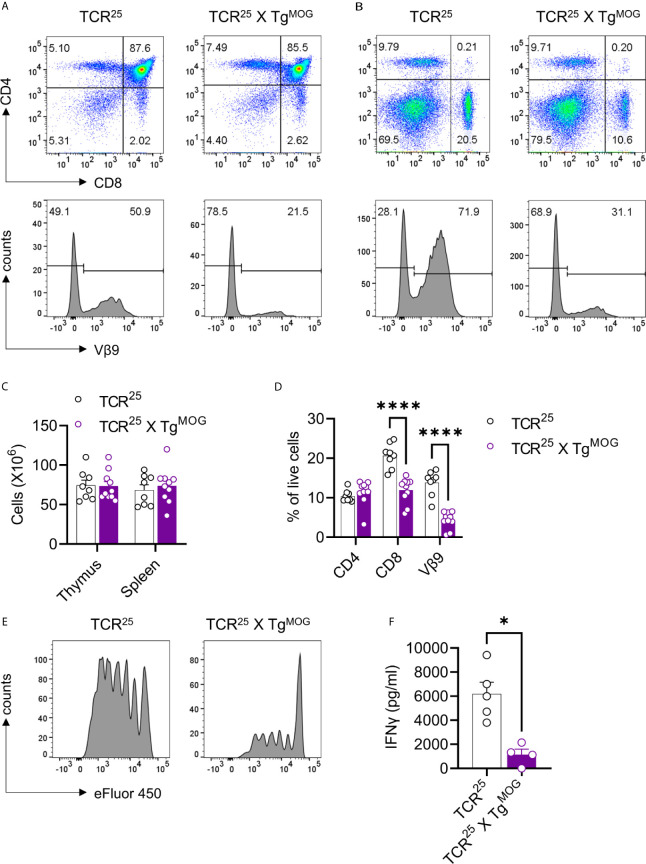
Decreased MOG specific CD8^+^ T cells in TCR^25^ X Tg^MOG^ mice. **(A, B)**, Flow cytometry analysis of thymus **(A)** and spleen **(B)** from TCR^25^ and TCR^25^ X Tg^MOG^ mice. The expression pattern of CD4 and CD8 is shown (upper panel). Lower panel histograms show transgenic Vβ9^+^ TCR on gated CD8 single-positive cells. Representative data from more than three independent experiments is shown. **(C)**, Absolute numbers of immune cells from thymus and spleen. The combined data from two independent experiments is shown. Each circle represents an individual mouse. n = 8 – 10 per group. **(D)**, Frequencies of CD4^+^, CD8^+,^ and Vβ9^+^ T cell populations in the spleen of TCR^25^ and TCR^25^ X Tg^MOG^ mice. The combined data from two independent experiments is shown. Each circle represents an individual mouse. n = 8 – 10 per group ****P < 0.0001 (Two-way ANOVA). **(E)**, Proliferation of CD8^+^ T cells from TCR^25^ or TCR^25^ X Tg^MOG^ mice in response to MOG_206-214_ (2 µg/ml). Histograms of cell proliferation dye e450 fluorescence on the gated CD8^+^ T cells are shown. Representative data from 2 experiments are shown. **(F)** IFNγ secretion by splenocytes in response to MOG_206-214_ (20 µg/ml). Each circle represents an individual mouse. n = 4 – 5 per group *P = 0.0159 (Mann-Whitney’s U-test, two-tailed).

We sought to determine the impact of MOG expression on CD8^+^ and CD4^+^ T cell tolerance. To study the impact of MOG-expression on MOG-specific CD8^+^ T cells, we generated TCR transgenic mice (TCR^25^) recognizing intracellular MOG_206-214_ epitope. This new transgenic mouse strain express Vα5/Vβ9 TCR that recognizes MOG_206-214_ in the context of H2-Kb **(** **Supplementary Figures 3A–D** **)**. Analysis of thymus and spleen of TCR^25^ mice showed an expression of Vβ9 TCR exclusively in the CD8^+^ T cells and these cells react to MOG_206-214_ **(** **Supplementary Figure 3E** **)**. We crossed TCR^25^ mice with Tg^MOG^ mice and observed more than 50% reduction in the transgenic Vβ9^+^ CD8^+^ T cells in the thymus and spleen of TCR^25^ x Tg^MOG^ double-transgenic mice **(** **Figures 4A–D** **)**. The remaining CD8^+^ T cells from TCR^25^ x Tg^MOG^ mice showed a reduced proliferative response and IFNγ secretion after MOG_206-214_ stimulation when compared to single transgenic TCR^25^ littermates (**Figures 4E, F**). These results suggest that ubiquitous expression of MOG results in a deletional CD8^+^ T cell tolerance.

Since MOG-reactive CD4^+^ T cells are the most important cell type for the induction of the neuroinflammatory disease experimental autoimmune encephalomyelitis (EAE), we examined the influence of MOG-expression on CD4^+^ T cell tolerance. To this end, we crossed the Tg^MOG^ transgenic mice with TCR^MOG^ (2D2) (14) and TCR^ova^ (OT-II) (18) transgenic mice, which show a high frequency of MOG- and Ova-specific CD4^+^ T cells, respectively, in their lymphoid organs. About 4 - 15% of 2D2 mice spontaneously develop autoimmune neuritis and paralytic EAE symptoms (14, 16). However, 2D2 x Tg^MOG^ and OT-II x Tg^MOG^ double-transgenic mice remained free of any clinical signs during a >6-month observation period.

**Figure 5:**
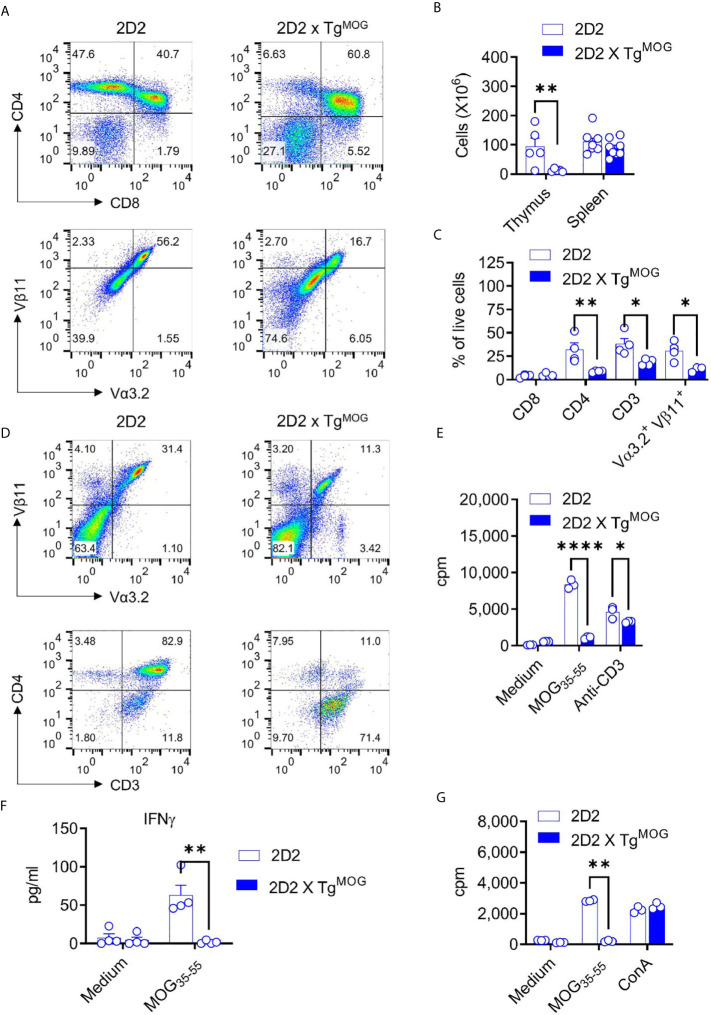
MOG-specific CD4^+^ T cell - tolerance in 2D2 x Tg^MOG^ mice. **(A)**, Flow cytometry analysis of thymus from 2D2 and 2D2 X Tg^MOG^ littermates. Representative plots are shown. **(B)**, Cellularity of thymus and spleen from 2D2 and 2D2 X Tg^MOG^ littermates. The combined data from two independent experiments is shown. Each circle represents an individual mouse. n = 5 - 8 mice per group. **P = 0.0021 (Two-way ANOVA). **(C)**, Immune cell composition of the spleen from 2D2 and 2D2 X Tg^MOG^ mice. Frequencies of CD4^+^, CD8^+^, CD3^+^, and transgenic TCR Vα3.2^+^Vβ11^+^ cells in the live gate are shown. Representative data from two independent experiments is shown. Each circle represents an individual mouse. n = 3 – 4 mice per group. **P = 0.0037, *P < 0.02 (Two-way ANOVA). **(D)**, Analysis of CD4 and CD3 expression in transgenic TCR Vα3.2^+^Vβ11^+^ cells. Representative data from more than two independent experiments is shown. **(E)**, Proliferation of splenocytes from 2D2 and 2D2 X Tg^MOG^ mice. Representative data from two independent experiments is shown. Each circle represents an individual replicate. ****P < 0.0001, *P = 0.0223 (Two-way ANOVA). **(F)**, IFNγ secretion by splenocytes in response to MOG_35-55_. Representative data from two independent experiments is shown. Each circle represents an individual mouse. n = 4 per group. **P = 0.0026 (Two-way ANOVA). **(G)**, Proliferation of splenocytes from MOG_35-55_ immunized 2D2 and 2D2 X Tg^MOG^ mice. Representative data from two independent experiments is shown. Each circle represents an individual replicate. **P = 0.0012, *P = 0.0348 (Two-way ANOVA).

Thymic T cell development was severely impaired in 2D2 x Tg^MOG^ double-transgenic mice which showed reduced cellularity in the thymus compared to 2D2 single-transgenic mice **(** **Figures 5A, B** **)**. However, cell numbers of the spleen were similar among both groups **(** **Figure 5B** **)**. Flow cytometric analysis demonstrated a significant reduction, but not a complete loss, of CD4^+^ single-positive cells in the thymus of 2D2 x Tg^MOG^ double-transgenic mice compared to 2D2 single-transgenic littermates **(** **Figures 5A, B** **)**. In contrast, we noted a substantial increase of double-negative (DN) (CD4^–^CD8^–^) thymocytes in 2D2 x Tg^MOG^ mice indicating a strong negative selection owing to the expression of MOG. Most of these DN cells represent post DN4 cells and are not the bone marrow precursor cells population indicated by the loss of CD24 and expression of CD3 and TCR (data not shown). Consistent with the cell numbers in the spleen, we found TCR Vα3.2^+^ Vβ11^+^ transgenic T cells in the 2D2 x Tg^MOG^ double-transgenic mice **(** **Figures 5C, D** **)**. Besides, we observed that Vα3.2^+^Vβ11^+^ T cells that have escaped thymic selection downregulated CD4 co-receptor on their surface **(** **Figure 5D** **)**. While more than 90% of Vα3.2^+^Vβ11^+^ T cells from 2D2 single-transgenic mice express the CD4 co-receptor, the majority of transgenic T cells do not express the CD4 co-receptor in 2D2 x Tg^MOG^ double-transgenic mice. The frequency of CD8^+^ T cells remained similar in double and single transgenic spleens **(** **Figure 5C** **)**. Although the frequencies of Foxp3^+^ regulatory T cells (Treg) in the thymus and spleen were increased, the absolute cell numbers were comparable between 2D2 x Tg^MOG^ and 2D2 transgenic mice (**Supplementary Figure 4**).

The substantial numbers of 2D2 transgenic T cells in the periphery of 2D2 x Tg^MOG^ double-transgenic mice may indicate that some MOG-specific, pathogenic T cells could have escaped negative selection in the thymus. Although we did not find marked differences in the frequencies of DN T cells in Tg^MOG^ mice, 2D2 transgenic mice that carry high (>90%) numbers of pathogenic CD4^+^ T cells could favor incomplete thymic selection. Hence, we stimulated splenocytes from 2D2 x Tg^MOG^ double-transgenic mice with MOG_35-55_ and assessed the proliferative response. A strong T cell proliferation was evident when antigen-independent TCR signaling was induced with anti-CD3 antibodies, however, 2D2 x Tg^MOG^ T cells did not respond to the MOG_35-55_ peptide **(** **Figure 5E** **)**. Likewise, cytokine secretion from 2D2 x Tg^MOG^ T cells in response to MOG35-55 peptide was also impaired (**Figure 5F**). The tolerance induced by the MOG expression in Tg^MOG^ mice does not cause generalized unresponsiveness, as the T cells from OT-II x Tg^MOG^ responded to the cognate peptide Ova_323-339_ in a similar way to their single transgenic counterparts (data not shown). Also, immunization of 2D2 x Tg^MOG^ double-transgenic mice with MOG_35-55_ together with complete Freund’s adjuvant (CFA) did not restore T cell responsiveness to MOG **(** **Figure 5G** **).**

### MOG-Expression in Bone Marrow Cells Induces Tolerance and Ameliorates Autoimmunity

**Figure 6:**
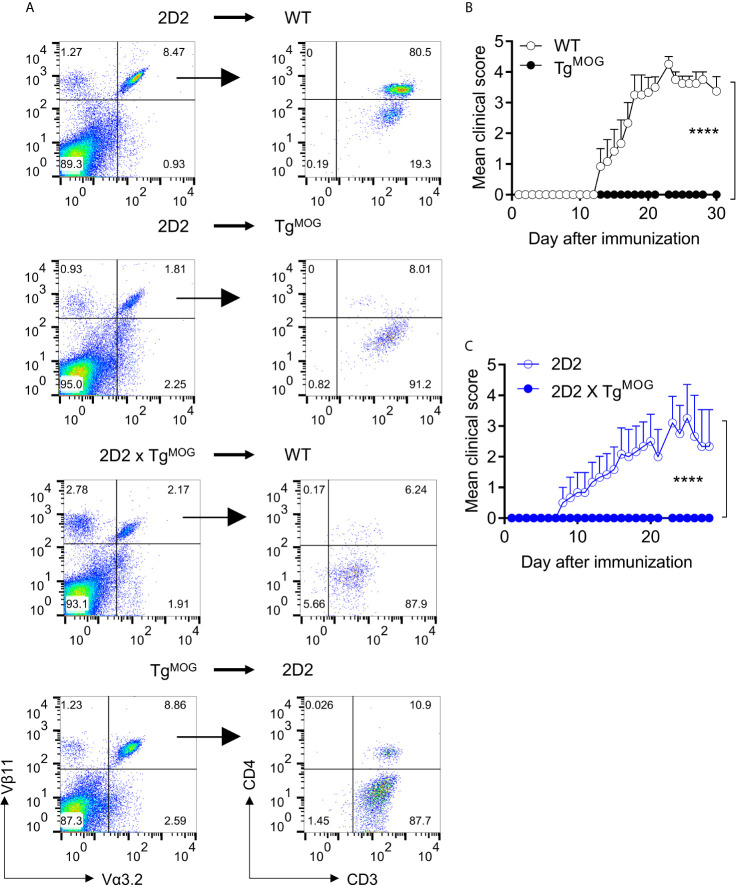
MOG expression in immune organs induces T cell tolerance and suppresses EAE. **(A)**, Analysis of CD4 and CD3 expression in transgenic TCR Vα3.2^+^Vβ11^+^ cells after bone marrow transplantation. Representative plots are shown. **(B)**, Mean EAE clinical scores in WT (n = 6) or Tg^MOG^ (n = 6) mice immunized with MOG_35-55_. **(C)**, Mean EAE clinical scores in 2D2 (n = 6) or 2D2 X Tg^MOG^ (n = 4) mice immunized with MOG_35-55_. **(B, C),** Representative data from two independent experiments is shown. ****P < 0.0001 (Two-way ANOVA).

The above data show that transgenic expression of MOG in thymic bone marrow-derived cells or stromal cells induces both deletions of MOG-specific CD4^+^ T cells as well as downregulation of CD4 co-receptor. To understand whether the expression of MOG in bone marrow cells is sufficient to induce tolerance phenotype, we transplanted MOG-expressing bone marrow cells from Tg^MOG^ mice into 2D2 mice. Transfer of MOG-expressing bone marrow cells was sufficient to induce CD4 downregulation similar to 2D2 x Tg^MOG^ mice **(** **Figure 6A** **).** Similarly, the transplantation of 2D2 bone marrow cells into Tg^MOG^ mice or 2D2 x Tg^MOG^ bone marrow cells into WT mice resulted in the impaired development of 2D2 T cells and the few cells that have escaped the selection largely devoid of CD4 co-receptor expression (**Figure 6A**). Finally, we tested the impact of transgenic MOG expression outside the CNS on the development of EAE induced by immunization with MOG_35-55_ peptide. While immunization with MOG_35-55_ peptide produced severe paralytic disease at high incidence in non-transgenic littermates, the Tg^MOG^ transgenic littermates were completely resistant to EAE induction **(** **Figure 6B** **).** Similarly, immunization of 2D2 x Tg^MOG^ mice also did not produce EAE symptoms in these mice **(** **Figure 6C** **)**. Taken together, we conclude that the expression of MOG in the periphery, especially in the bone marrow cells, induces a potent and long-lasting tolerance to MOG-specific lymphocytes.

## Discussion

This study has addressed whether antigen-specific immune tolerance to a myelin autoantigen can be established by the expression of the autoantigen outside the CNS. We engineered the immune cells to express MOG, which is normally expressed exclusively in the CNS and is not found in the classical primary and secondary lymphoid organs. We found that ectopic expression of MOG in the lymphoid organs led to the deletion of MOG-specific CD4, CD8, and B lymphocytes. Most of the MOG-specific lymphocytes developing in the TCR and BCR transgenic mice were deleted and few that have escaped remained unresponsive to the antigen stimulation. Eventually, this led to a life-long tolerance to MOG-induced autoimmune disease. Our finding shows that enforced expression of autoantigens in primary lymphoid organs is an attractive strategy for the treatment of specific autoantigen-driven diseases.

Antigen-specific tolerization approaches are an ideal goal for the management of autoimmune diseases. However, many autoimmune diseases are complex and are probably driven by many autoantigens. This is especially true for MS, a classical autoimmune inflammatory disorder of the CNS. Although T and B cell responses against numerous myelin and neuronal antigens have been identified in MS patients, none is shown to be a specific driver of MS (19). The currently successful therapies used in MS patients are not specific and employ approaches to either deplete immune cells or prevent the entry of lymphocytes to the CNS. The problem with these approaches is the suppression of normal immune responses in case B cell depleting anti-CD20 antibodies (20) or defective immune surveillance of the CNS leading to fatal diseases like progressive multifocal leukoencephalopathy (PML) in case of anti-VLA4 antibodies (21). Early antigen-specific therapeutic approaches like altered peptide ligand (APL) which are designed to suppress T cell reactivity against MBP was highly attractive, but clinical trials with APL enhanced the disease activity (22, 23). It should be noted that different MS patients may have different autoimmune reactivity and targeting one autoantigen MBP may not be ideal in a heterogeneous group of MS patients.

Some years ago, autoantibodies against astrocyte antigen AQP4 has been discovered in a subset of patients with neuromyelitis optica spectrum disorders (NMOSD) (24). Recently, a new neurological condition associated with pathogenic serum antibodies against MOG which is distinct from MS and AQP4 antibody-positive NMOSD has also been identified (25). While the cellular composition of CNS lesions in MOG-antibody disease patients is currently unknown, there is a possibility that MOG-driven T cell responses are responsible for the generation of MOG autoantibodies.

The identification of specific autoantigens in a subgroup of MS patients raises the possibility that antigen-specific therapeutic approaches likely benefit those patients. Some of the antigen-specific therapeutic approaches that have been previously tried in experimental models of MS include injection of fused autoantigen peptides (26, 27), peptide nanoparticles (28), peptide-MHC coated nanoparticles (29), DNA (30, 31), or peptide mRNA (32). Despite their efficiency in suppressing neuroinflammation, the long-term efficiency of those approaches is not known. Most importantly most of these approaches were targeted against CD4^+^ T cells while it is known that CD8^+^ T cells and B cells are also crucially important for MS disease pathogenesis. Also, as many epitopes within the same autoantigen can be recognized by the T cells and B cells, peptide epitope approaches will be of limited value.

An alternative approach is to harness the natural tolerance induction process employed by our immune system to purge autoreactive cells permanently from the repertoire. To test the feasibility of this approach, we enforced MOG expression in the immune system by transgenic technology. This resulted in higher expression of MOG protein in all cells of the immune system in the spleen and lymph nodes. The expression of MOG in the thymus and bone marrow immune cells was variable but was sufficient to purge emerging autoreactive T cells or B cells. Since it is difficult to enumerate MOG-specific T and B cells in naïve mice, we crossed MOG-expressing mice with MOG-specific CD4^+^, CD8^+^, and B cell receptor transgenic mice. Similar to other model systems (33, 34), MOG expression resulted in a deletion of antigen-specific B cells and T cells. Some of the MOG-reactive T cells have escaped the thymic selection but had reduced TCR expression and downregulation of CD4 co-receptor. Similar downregulation of TCR and CD4 molecules has been observed in antigen-TCR double transgenic model systems (34, 35). However, the escaped T cells were unresponsive to *in vitro* stimulation with a cognate antigen.

Finally, we noted a comparable deletion of T cells by a transfer of bone marrow cells expressing MOG, in line with previous reports which showed T and B cell deletion upon transfer of retrovirally transduced MOG in bone marrow stem cells (36, 37). AHSCT has been performed in MS patients to reset the immune repertoire, but dominant CD8^+^ clones were not completely removed after reconstitution (38). This poses a greater risk of disease relapses and it is unknown if the T cells targeting new epitopes of the same antigen will emerge from the reconstituted immune system. Our results suggest that expressing autoantigens before transplantation likely have a favorable long-term beneficial outcome. Although we largely observed deletion of antigen-specific lymphocytes, some autoreactive lymphocytes may escape the selection process in the primary lymphoid organs. But the expression of autoantigens in bone marrow stem cells likely results in the expression of autoantigen in all immune cell populations that occupy secondary lymphoid organs. This will result in the enforcement of additional tolerance induction mechanisms. Indeed, we observed deletion of MOG-specific T cells by a transfer of MOG-expressing immature dendritic cells suggesting that if autoreactive lymphocytes escape negative selection they will be further subjected to additional tolerance mechanisms.

## Conclusions

The data presented here show that long-lasting MOG-specific tolerance to T and B cells can be established by ectopic expression in immune cells. This observation has important implications to re-establish tolerance not only in MOG-driven neurological disease but also for the therapy of other autoimmune diseases driven by specific autoantigens.

## Data Availability Statement

The original contributions presented in the study are included in the article/**Supplementary Material**. Further inquiries can be directed to the corresponding author.

## Ethics Statement**

All animal experiments were performed according to the institutional guides and the protocols were approved by Regierung von Oberbayern (Munich, Germany).

## Author Contributions

S-YN and GK performed the experiments. GK wrote the manuscript. All authors contributed to the article and approved the submitted version.

## Funding

This work was funded by the Max Planck Society. GK is further supported by the European Research Council starting grant (GAMES; 635617), German research foundation (DFG) SFB TR-128 (Project A1). None of the funding sources had an influence on the study design, data collection, analysis, interpretation, or the writing of the manuscript.

## Conflict of Interest

The authors declare that the research was conducted in the absence of any commercial or financial relationships that could be construed as a potential conflict of interest.

## Supplementary Material

Supplementary Figure 1 Immune cell composition of the lymph node and spleen of Tg^MOG^ and WT mice. **A,** Frequencies of CD19^+^, CD4^+^, CD8^+^, CD11c^+^, CD11b^+,^ and Gr-1^+^ cells in the live gated lymph node cells are shown. **B,** Activation marker expression in splenic CD8^+^ T cells. Frequencies of CD62L^low^, CD69^+^, PD-1^+^ cells (left) or mean fluorescence intensity (MFI) of CD44 expression (right) in gated CD8^+^ cells are shown. **C,** Frequencies of specific TCR α and β chain expressing T cells in the spleen were determined by flow cytometry. **A–C,** The combined data from two independent experiments is shown. Each circle represents an individual mouse. n = 4 per group.

Supplementary Figure 2 MOG-specific B cells are deleted in Tg^MOG^ mice. eFluor450 labeled splenocytes from IgH^MOG^ mice were transferred into Tg^MOG^ or WT littermates and the spleen of the recipient animals were analyzed after 5 days. **A,** Flow cytometry analysis of spleen from Tg^MOG^ or WT recipients. Dot plots show anti-IgM^a^ and B220 staining (top panel) or recombinant MOG with B220 (bottom panel) gated on eFluor450 positive cells. Representative data from 4 individual mice are shown. **B,** Frequencies of eFluor450 and IgM^a^ or MOG binding B cell population in the spleen. Each circle represents an individual mouse. n = 4 mice per group. ****P < 0.0001, ***P = 0.0007 (Two-way ANOVA).

Supplementary Figure 3 Characterization of TCR^25^ mice. **A, B,** Flow cytometry analysis of thymus **(A)** and spleen **(B)** from WT and TCR^25^ mice (upper panel). The expression pattern of CD4 and CD8 is shown (upper panel). Lower panel histograms show transgenic Vβ9^+^ TCR on gated CD8 single-positive cells. Representative data from more than three independent experiments is shown. **C,** Absolute numbers of immune cells from thymus and spleen. Each circle represents an individual mouse. n = 3 per group. **D,** Frequencies of CD4^+^, CD8^+,^ and Vβ9^+^ T cell populations in the spleen. Each circle represents an individual mouse. n = 3 per group. *P = 0.0158, **P = 0.0078, ***P = 0.0008 (Two-way ANOVA). **E,** Proliferation of TCR^25^ mouse CD8^+^ T cells in response MOG_206-214._ Histograms of cell proliferation dye e450 fluorescence on the gated CD8^+^ T cells are shown. Representative data from 3 experiments are shown.

Supplementary Figure 4 Expression of Foxp3 in the thymus and spleen of 2D2 or 2D2 X Tg^MOG^ mice. **A, B,** Flow cytometry analysis of thymus **(A)** and spleen **(B)** from 2D2 and 2D2 X Tg^MOG^ littermates. Representative plots are shown. **C, D,** Absolute cell numbers **(C)**, and frequencies **(D)** of CD4^+^ and Foxp3^+^ cells in the thymus and spleen from 2D2 and 2D2 X Tg^MOG^ littermates. Each circle represents an individual mouse. n = 2 - 4 mice per group. **P = 0.0021, ****P < 0.0001 (Two-way ANOVA).

Supplementary Figure 5 Gating scheme. A sample gating strategy for defining different immune populations.

## Abbreviations

APL, Altered peptide ligand; AQP4, Aquaporin 4; AHSCT, Autologous hematopoietic stem cell transplantation; BCR, B cell receptor; BM, Bone marrow; CNS, Central Nervous System; cDNA, Complementary Deoxyribonucleic Acid; ConA, Concanavalin A; CFA, Complete Freund’s adjuvant; Cpm, Counts per minute; DN, Double negative; EAE, Experimental Autoimmune Encephalomyelitis; ELISA, Enzyme linked immunosorbent assay; LPS, Lipopolysaccharide; LN, Lymph node; MOG, Myelin oligodendrocyte glycoprotein; NMOSD, Neuromyelitis optica spectrum disorders; MS, Multiple Sclerosis; OD, Optical density; PMA, Phorbol 12-myristate 13-acetate; PML, Progressive multifocal leukoencephalopathy; TCR, T cell receptor.

## References

[1] Rosenblum MD Remedios KA Abbas AK. Mechanisms of Human Autoimmunity. J Clin Invest (2015) 125:2228–33. 10.1172/JCI78088 PMC4518692 25893595

[2] Kyewski B Klein L. Central Role for Central Tolerance. Annu Rev Immunol (2006) 24:571–606. 10.1146/annurev.immunol.23.021704.115601 16551260

[3] Serra P Santamaria P. Antigen-Specific Therapeutic Approaches for Autoimmunity. Nat Biotechnol (2019) 37:238–51. 10.1038/s41587-019-0015-4 30804535

[4] Wynford-Thomas R Jacob A Tomassini V. Neurological Update: MOG Antibody Disease. J Neurol (2018) 266:1280–6. 10.1007/s00415-018-9122-2 PMC6469662 30569382

[5] Spadaro M Winklmeier S Beltrán E Macrini C Höftberger R Schuh E. Pathogenicity of Human Antibodies Against Myelin Oligodendrocyte Glycoprotein. Ann Neurol (2018) 84:315–28. 10.1002/ana.25291 30014603

[6] Kyewski B Derbinski J. Self-Representation in the Thymus: An Extended View. Nat Rev Immunol (2004) 4:688–98. 10.1038/nri1436 15343368

[7] Klein L Klugmann M Nave KA Tuohy VK Kyewski BA. Shaping of the Autoreactive T Cell Repertoire by a Splice Variant of Self Protein Expressed in Thymic Epithelial Cells. Nat Med (2000) 6:56–61. 10.1038/71540 10613824

[8] Lucca LE Axisa P-P Aloulou M Perals C Ramadan A Rufas P. Myelin Oligodendrocyte Glycoprotein Induces Incomplete Tolerance of CD4+ T Cells Specific for Both a Myelin and a Neuronal Self-Antigen in Mice. Eur J Immunol (2016) 46:2247–59. 10.1002/eji.201646416 27334749

[9] Krishnamoorthy G Wekerle H. Eae: An Immunologist’s Magic Eye. Eur J Immunol (2009) 39:2031–5. 10.1002/eji.200939568 19672898

[10] Johns TG Bernard CC. The Structure and Function of Myelin Oligodendrocyte Glycoprotein. J Neurochem (1999) 72:1–9. 10.1046/j.1471-4159.1999.0720001.x 9886048

[11] Pagany M Jagodic M Schubart A Pham-Dinh D Bachelin C Baron van EA. Myelin Oligodendrocyte Glycoprotein is Expressed in the Peripheral Nervous System of Rodents and Primates. Neurosci Lett (2003) 350:165–8. 10.1016/S0304-3940(03)00899-1 14550920

[12] Burt RK Traynor A. Hematopoietic Stem Cell Therapy of Autoimmune Diseases. Curr Opin Hematol (1998) 5:472–7. 10.1097/00062752-199811000-00020 9814658

[13] Pircher H Mak TW Ballhausen W Rüedi E Hengartner H Zinkernagel RM. T Cell Tolerance to Mls a Encoded Antigens in T Cell Receptor Vß8.1 Chain Transgenic Mice. EMBO J (1989) 8:719–27. 10.1002/j.1460-2075.1989.tb03431.x PMC400867 2524380

[14] Bettelli E Pagany M Weiner HL Linington C Sobel RA Kuchroo VK. Myelin Oligodendrocyte Glycoprotein-Specific T Cell Receptor Transgenic Mice Develop Spontaneous Autoimmune Optic Neuritis. J Exp Med (2003) 197:1073–81. 10.1084/jem.20021603 PMC2193967 12732654

[15] Litzenburger T Fässler R Bauer J Lassmann H Linington C Wekerle H. B Lymphocytes Producing Demyelinating Autoantibodies: Development and Function in Gene-Targeted Transgenic Mice. J Exp Med (1998) 188:169–80. 10.1084/jem.188.1.169 PMC2525547 9653093

[16] Krishnamoorthy G Saxena A Mars LT Domingues HS Mentele R Ben-Nun A. Myelin Specific T Cells Also Recognize Neuronal Autoantigen in a Transgenic Mouse Model of Multiple Sclerosis. Nat Med (2009) 15:626–32. 10.1038/nm.1975 19483694

[17] Litzenburger T Blüthmann H Morales P Pham-Dinh D Dautigny A Wekerle H. Development of MOG Autoreactive Transgenic B Lymphocytes: Receptor Editing In Vivo Following Encounter of a Self-Antigen Distinct From MOG. J Immunol (2000) 165:5360–6. 10.4049/jimmunol.165.9.5360 11046072

[18] Barnden MJ Allison J Heath WR Carbone FR. Defective TCR Expression in Transgenic Mice Constructed Using cDNA-based α- and ß-Chain Genes Under the Control of Heterologous Regulatory Elements. Immunol Cell Biol (1998) 76:34–40. 10.1046/j.1440-1711.1998.00709.x 9553774

[19] Sospedra M Martin R. Immunology of Multiple Sclerosis. Semin Neurol (2016) 36:115–27. 10.1055/s-0036-1579739 27116718

[20] Ancau M Berthele A Hemmer B. CD20 Monoclonal Antibodies for the Treatment of Multiple Sclerosis: Up-to-Date. Expert Opin Biol Ther (2019) 19:829–43. 10.1080/14712598.2019.1611778 31027436

[21] Kleinschmidt-DeMasters BK Tyler KL. Progressive Multifocal Leukoencephalopathy Complicating Treatment With Natalizumab and Interferon ß-1a for Multiple Sclerosis. N Engl J Med (2005) 353:369–74. 10.1056/NEJMoa051782 15947079

[22] Bielekova B Goodwin B Richert N Cortese I Kondo T Afshar G. Encephalitogenic Potential of the Myelin Basic Protein Peptide (Amino Acids 83-99) in Multiple Sclerosis: Results of a Phase II Clinical Trial With an Altered Peptide Ligand. Nat Med (2000) 6:1167–75. 10.1038/80516 11017150

[23] Kappos L Comi G Panitch H Oger J Antel J Conlon P. The, Induction of a non-Encephalitogenic Th2 Autoimmune Response in Multiple Sclerosis After Administration of an Altered Peptide Ligand in a Placebo Controlled, Randomized Phase II Trial. Nat Med (2000) 6:1176–82. 10.1038/80525 11017151

[24] Lennon VA Kryzer TJ Pittock SJ Verkman AS Hinson SR. Igg Marker of Optic-Spinal Multiple Sclerosis Binds to the Aquaporin-4 Water Channel. J Exp Med (2005) 202:473–7. 10.1084/jem.20050304 PMC2212860 16087714

[25] Reindl M Waters P. Myelin Oligodendrocyte Glycoprotein Antibodies in Neurological Disease. Nat Rev Neurol (2019) 15:89–102. 10.1038/s41582-018-0112-x 30559466

[26] Kaushansky N Kaminitz A Allouche-Arnon H Ben-Nun A. Modulation of MS-like Disease by a Multi Epitope Protein is Mediated by Induction of CD11c^+^CD11b^+^Gr1^+^ Myeloid-Derived Dendritic Cells. J Neuroimmunol (2019) 333:476953. 10.1016/j.jneuroim.2019.04.013 31108399

[27] Zhong MC Kerlero de Rosbo N Ben-Nun A. Multiantigen/Multiepitope-Directed Immune-Specific Suppression of “Complex Autoimmune Encephalomyelitis” by a Novel Protein Product of a Synthetic Gene. J Clin Invest (2002) 110:81–90. 10.1172/JCI0215692 12093891 PMC151033

[28] Kenison JE Jhaveri A Li Z Khadse N Tjon E Tezza S. Tolerogenic Nanoparticles Suppress Central Nervous System Inflammation. Proc Natl Acad Sci USA (2020) 117:32017–28. 10.1073/pnas.2016451117 PMC7749362 33239445

[29] Clemente-Casares X Blanco J Ambalavanan P Yamanouchi J Singha S Fandos C. Expanding Antigen-Specific Regulatory Networks to Treat Autoimmunity. Nature (2016) 530:434–40. 10.1038/nature16962 26886799

[30] Fissolo N Costa C Nurtdinov RN Bustamante MF Llombart V Mansilla MJ. Treatment With MOG-DNA Vaccines Induces CD4^+^CD25^+^FoxP3^+^ Regulatory T Cells and Up-Regulates Genes With Neuroprotective Functions in Experimental Autoimmune Encephalomyelitis. J Neuroinflamm (2012) 9139. 10.1186/1742-2094-9-139 PMC3464883 22727044

[31] Lobell A Weissert R Eltayeb S de Graaf KL Wefer J Storch MK. Suppressive DNA Vaccination in Myelin Oligodendrocyte Glycoprotein Peptide-Induced Experimental Autoimmune Encephalomyelitis Involves a T1-biased Immune Response. J Immunol (2003) 170:1806–13. 10.4049/jimmunol.170.4.1806 12574345

[32] Krienke C Kolb L Diken E Streuber M Kirchhoff S Bukur T. A Noninflammatory mRNA Vaccine for Treatment of Experimental Autoimmune Encephalomyelitis. Science (2021) 371:145–53. 10.1126/science.aay3638 33414215

[33] Nemazee D Russell D Arnold B Hämmerling GJ Allison J Miller JFAP. Clonal Deletion of Autospecific B Lymphocytes. Immunol Rev (1991) 122:117–32. 10.1111/j.1600-065X.1991.tb00600.x 1937539

[34] Teh HS Kishi H Scott B von Boehmer H. Deletion of Autospecific T Cells in T Cell Receptor (TcR) Transgenic Mice Spares Cells With Normal TcR Levels and Low Levels of CD8 Molecules. J Exp Med (1989) 169:795–806. 10.1084/jem.169.3.795 2494291 PMC2189279

[35] Schönrich G Kalinke U Momburg F Malissen M Schmitt-Verhulst AM Malissen B. Down-Regulation of T Cell Receptors on Self-Reactive T Cells as a Novel Mechanism for Extrathymic Tolerance Induction. Cell (1991) 65:293–304. 10.1016/0092-8674(91)90163-S 1849799

[36] Chung J-Y Figgett W Fairfax K Bernard C Chan J Toh B-H. Gene Therapy Delivery of Myelin Oligodendrocyte Glycoprotein (MOG) *Via* Hematopoietic Stem Cell Transfer Induces Mog-Specific B Cell Deletion. J Immunol (2014) 192:2593–601. 10.4049/jimmunol.1203563 24532581

[37] Chan J Ban EJ Chun KH Wang S Bäckström BT Bernard CCA. Transplantation of Bone Marrow Transduced to Express Self-Antigen Establishes Deletional Tolerance and Permanently Remits Autoimmune Disease. J Immunol (2008) 181:7571–80. 10.4049/jimmunol.181.11.7571 19017946

[38] Muraro PA Robins H Malhotra S Howell M Phippard D Desmarais C. T Cell Repertoire Following Autologous Stem Cell Transplantation for Multiple Sclerosis. J Clin Invest (2014) 124:1168–72. 10.1172/JCI71691 PMC3934160 24531550

