# Supplementary figures and images for: Targeted Expression of Myelin Autoantigen in the Periphery Induces Antigen-Specific T and B Cell Tolerance and Ameliorates Autoimmune Disease
Source: Front Immunol. 2021 Jun 2;12:668487. doi: 10.3389/fimmu.2021.668487 (PMC8206569; doi:10.3389/fimmu.2021.668487)

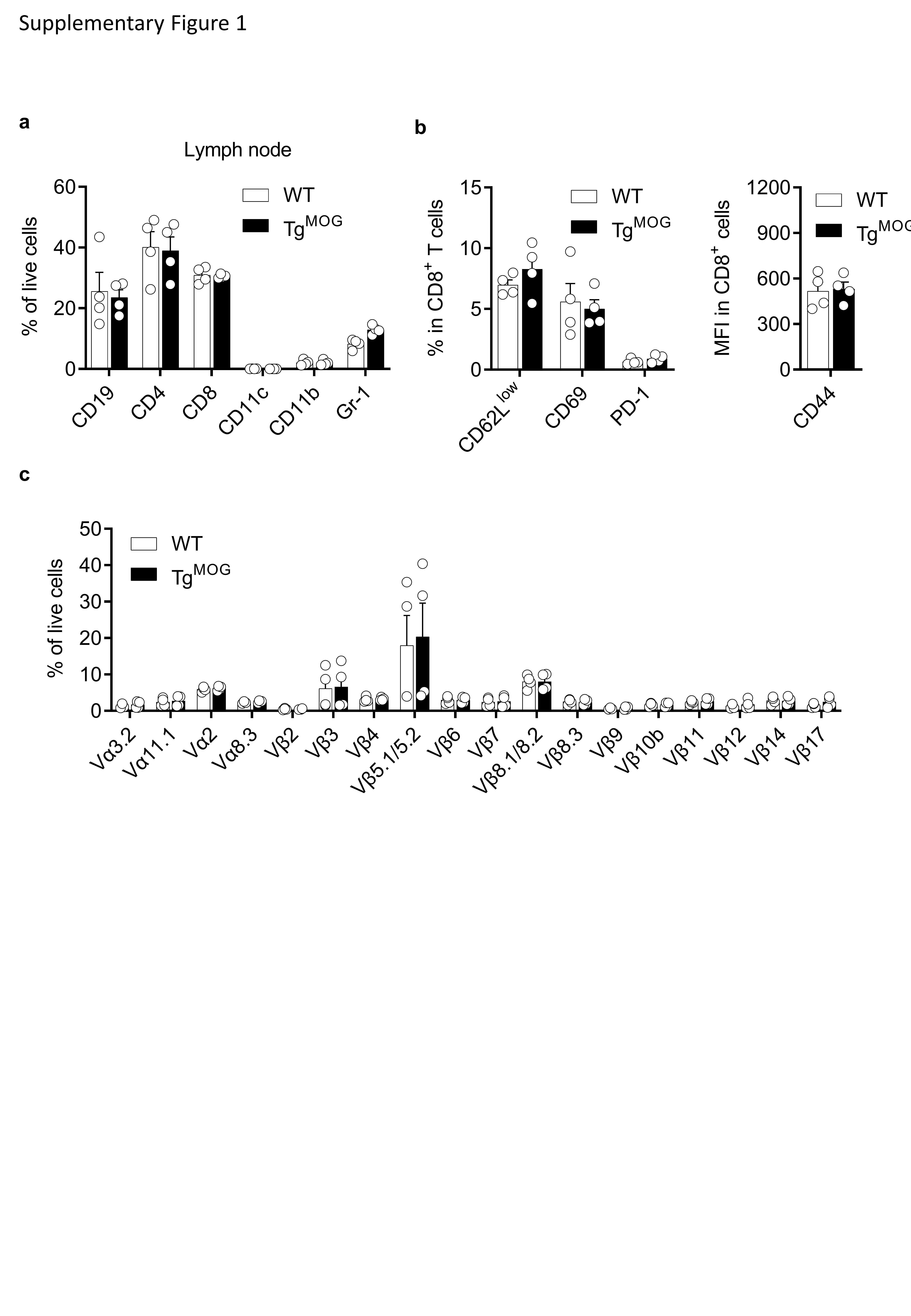

Supplement: Supplementary Figure 1 — Immune cell composition of the lymph node and spleen of TgMOG and WT mice. A, Frequencies of CD19+, CD4+, CD8+, CD11c+, CD11b+, and Gr-1+ cells in the live gated lymph node cells are shown. B, Activation marker expression in splenic CD8+ T cells. Frequencies of CD62Llow, CD69+, PD-1+ cells (left) or mean fluorescence intensity (MFI) of CD44 expression (right) in gated CD8+ cells are shown. C, Frequencies of specific TCR α and β chain expressing T cells in the spleen were determined by flow cytometry. A–C, The combined data from two independent experiments is shown. Each circle represents an individual mouse. n = 4 per group. [file Image_1.tif]

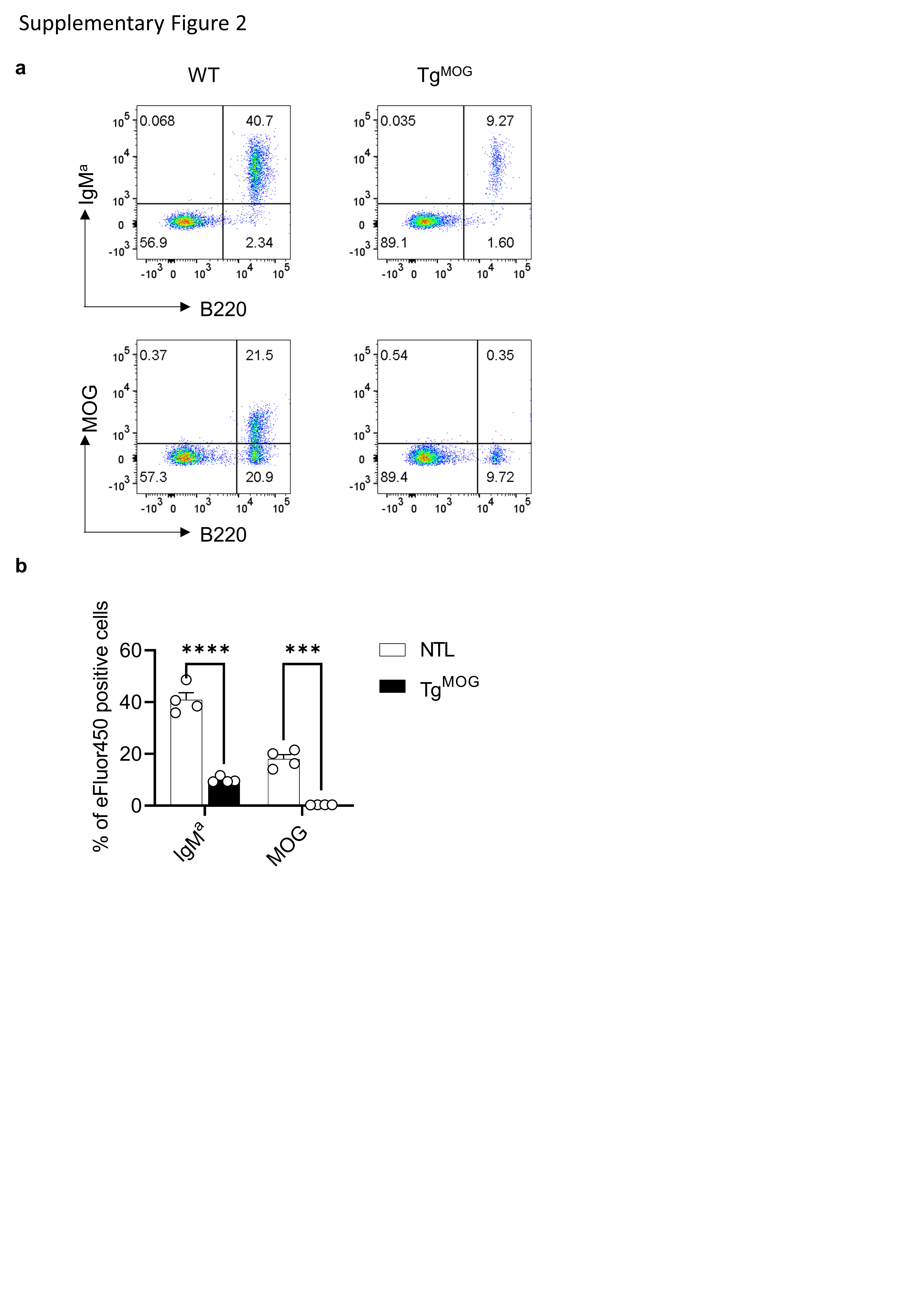

Supplement: Supplementary Figure 2 — MOG-specific B cells are deleted in TgMOG mice. eFluor450 labeled splenocytes from IgHMOG mice were transferred into TgMOG or WT littermates and the spleen of the recipient animals were analyzed after 5 days. A, Flow cytometry analysis of spleen from TgMOG or WT recipients. Dot plots show anti-IgMa and B220 staining (top panel) or recombinant MOG with B220 (bottom panel) gated on eFluor450 positive cells. Representative data from 4 individual mice are shown. B, Frequencies of eFluor450 and IgMa or MOG binding B cell population in the spleen. Each circle represents an individual mouse. n = 4 mice per group. ****P < 0.0001, ***P = 0.0007 (Two-way ANOVA). [file Image_2.tif]

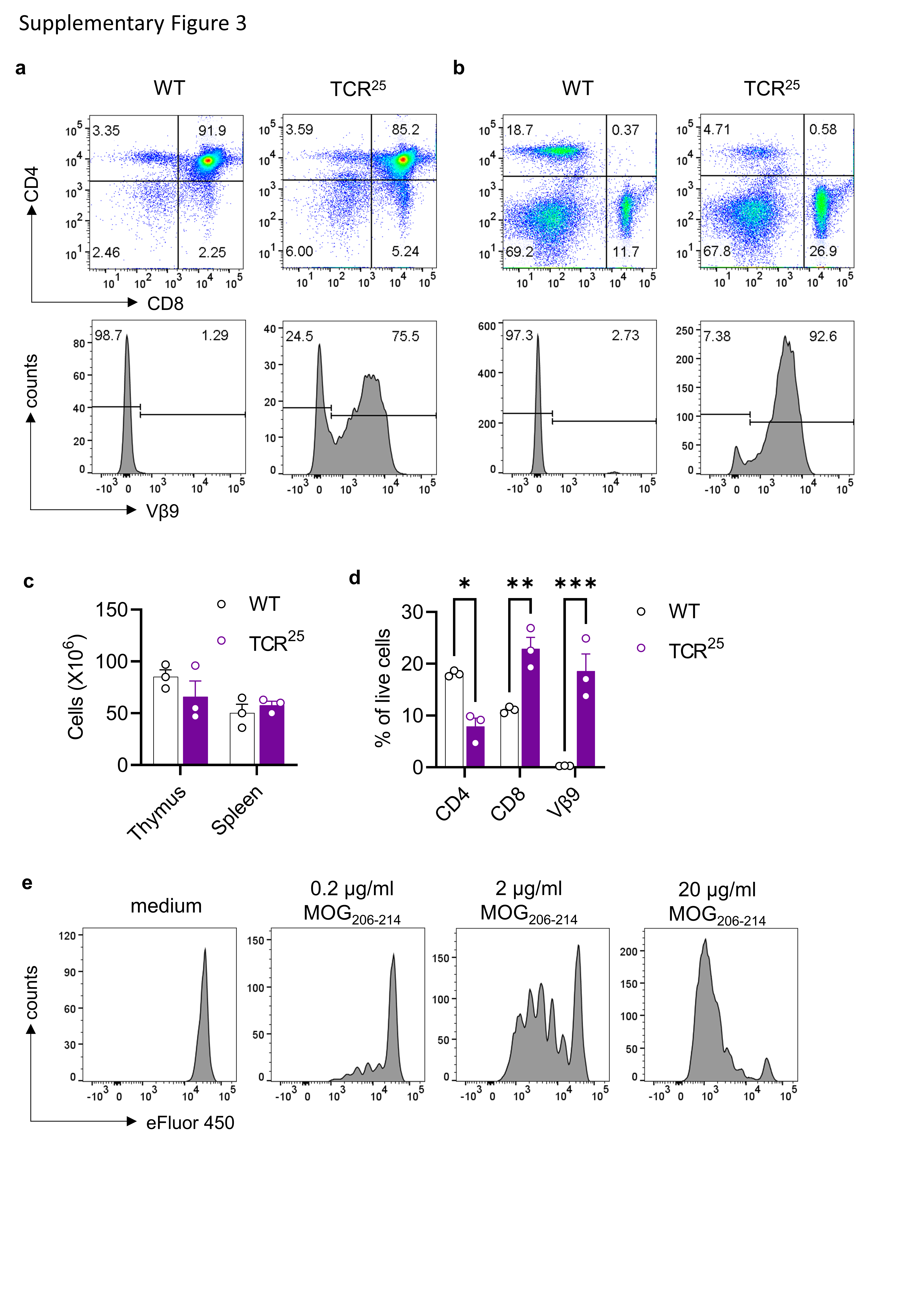

Supplement: Supplementary Figure 3 — Characterization of TCR25 mice. A, B, Flow cytometry analysis of thymus (A) and spleen (B) from WT and TCR25 mice (upper panel). The expression pattern of CD4 and CD8 is shown (upper panel). Lower panel histograms show transgenic Vβ9+ TCR on gated CD8 single-positive cells. Representative data from more than three independent experiments is shown. C, Absolute numbers of immune cells from thymus and spleen. Each circle represents an individual mouse. n = 3 per group. D, Frequencies of CD4+, CD8+, and Vβ9+ T cell populations in the spleen. Each circle represents an individual mouse. n = 3 per group. *P = 0.0158, **P = 0.0078, ***P = 0.0008 (Two-way ANOVA). E, Proliferation of TCR25 mouse CD8+ T cells in response MOG206-214. Histograms of cell proliferation dye e450 fluorescence on the gated CD8+ T cells are shown. Representative data from 3 experiments are shown. [file Image_3.tif]

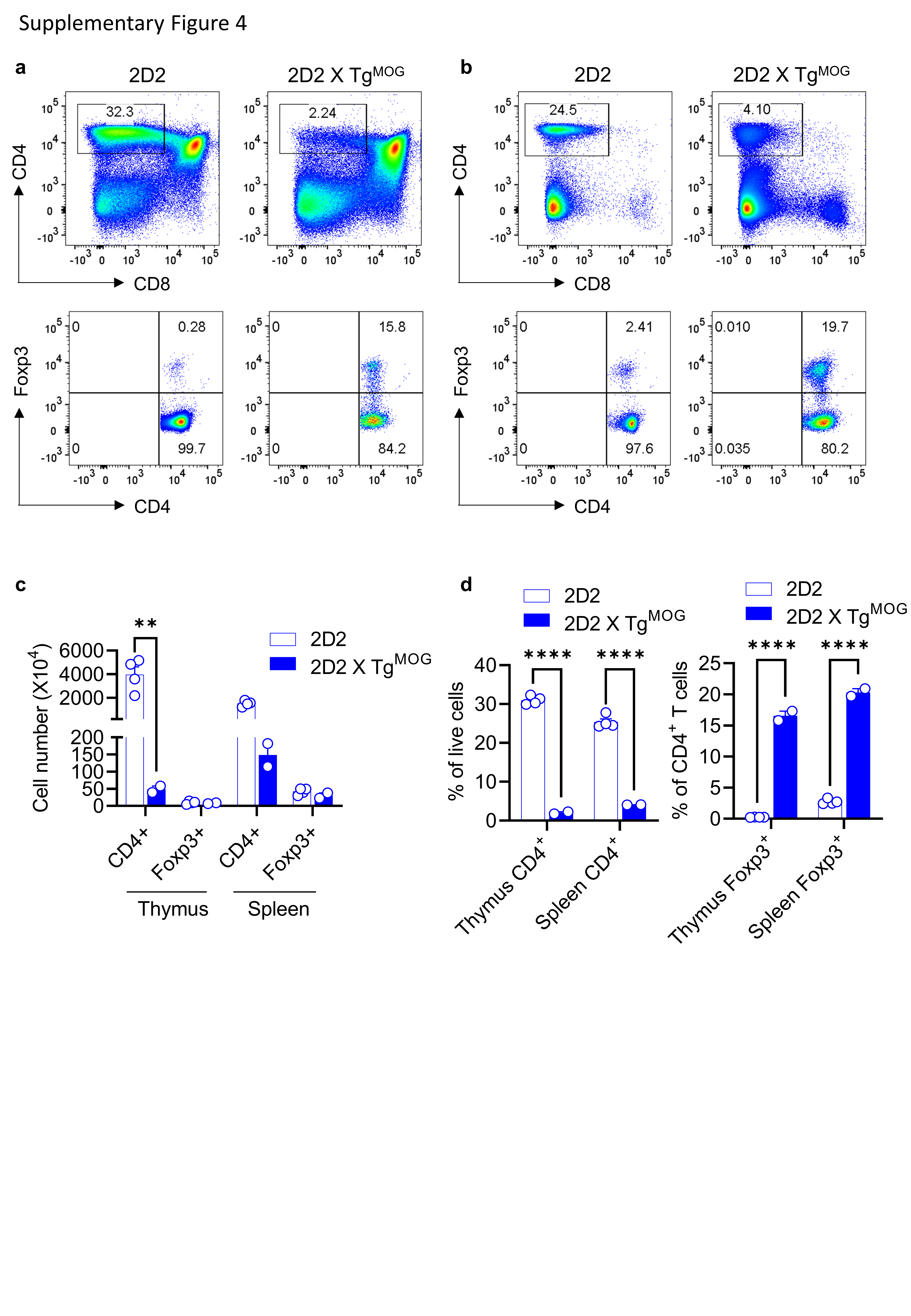

Supplement: Supplementary Figure 4 — Expression of Foxp3 in the thymus and spleen of 2D2 or 2D2 X TgMOG mice. A, B, Flow cytometry analysis of thymus (A) and spleen (B) from 2D2 and 2D2 X TgMOG littermates. Representative plots are shown. C, D, Absolute cell numbers (C), and frequencies (D) of CD4+ and Foxp3+ cells in the thymus and spleen from 2D2 and 2D2 X TgMOG littermates. Each circle represents an individual mouse. n = 2 - 4 mice per group. **P = 0.0021, ****P < 0.0001 (Two-way ANOVA). [file Image_4.tif]

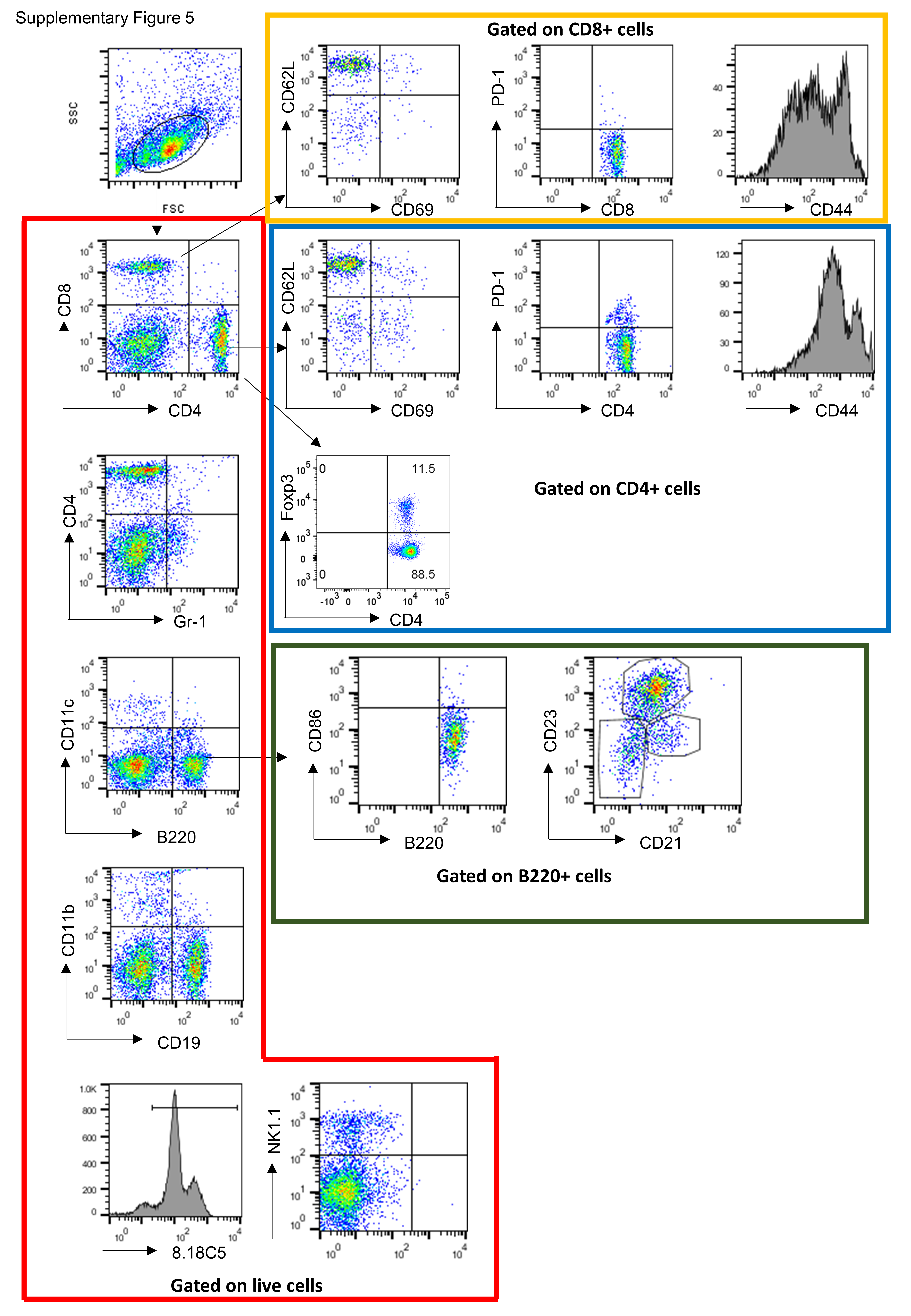

Supplement: Supplementary Figure 5 — Gating scheme. A sample gating strategy for defining different immune populations. [file Image_5.tif]
